# Supplementary material for: Evaluation of a mechanical lung model to test small animal whole body plethysmography
Source: Sci Rep. 2021 Aug 24;11:17099. doi: 10.1038/s41598-021-96355-y (PMC8384843; doi:10.1038/s41598-021-96355-y)
Supplement: Supplementary file 1 — Supplementary Information. [file 41598_2021_96355_MOESM1_ESM.docx]

**Supplementary Material:**


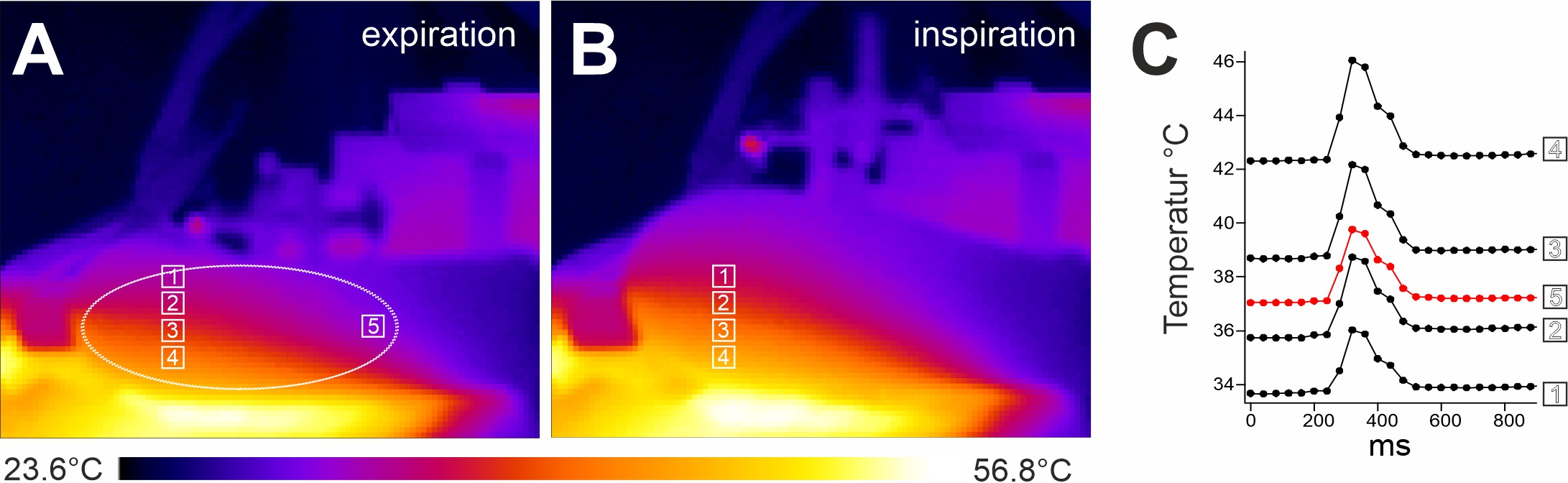


**Supplemental figure: Dummy model of the lung.** A-C: Measurement of temperature changes using a thermographic camera (FLIR A655sc) in expiration (A) and inspiration (B). Alterations during a breathing cycle are shown in (C) for 4 individual spots (1-4) and a region of interest (5, red trace) indicating an increase of the bulb temperature during inspiration.
